# Skeletal variation in extant species enables systematic identification of New Zealand’s large, subfossil diplodactylids

**DOI:** 10.1186/s12862-021-01808-7

**Published:** 2021-04-27

**Authors:** Lachie Scarsbrook, Emma Sherratt, Rodney A. Hitchmough, Nicolas J. Rawlence

**Affiliations:** 1 Otago Paleogenetics Laboratory, Department of Zoology, University of Otago, Dunedin, New Zealand; 2 School of Biological Sciences, The University of Adelaide, Adelaide, South Australia Australia; 3 Department of Conservation, Wellington, New Zealand

**Keywords:** Diplodactylidae, Ecomorphology, Geometric morphometrics, *Hoplodactylus duvaucelii*, Taxonomy

## Abstract

New Zealand’s diplodactylid geckos exhibit high species-level diversity, largely independent of discernible osteological changes. Consequently, systematic affinities of isolated skeletal elements (fossils) are primarily determined by comparisons of size, particularly in the identification of *Hoplodactylus duvaucelii*, New Zealand’s largest extant gecko species. Here, three-dimensional geometric morphometrics of maxillae (a common fossilized element) was used to determine whether consistent shape and size differences exist between genera, and if cryptic extinctions have occurred in subfossil *‘Hoplodactylus cf. duvaucelii’*. Sampling included 13 diplodactylid species from five genera, and 11 Holocene subfossil ‘*H. cf. duvaucelii*’ individuals. We found phylogenetic history was the most important predictor of maxilla morphology among extant diplodactylid genera. Size comparisons could only differentiate *Hoplodactylus* from other genera, with the remaining genera exhibiting variable degrees of overlap. Six subfossils were positively identified as *H. duvaucelii*, confirming their proposed Holocene distribution throughout New Zealand. Conversely, five subfossils showed no clear affinities with any modern diplodactylid genera, implying either increased morphological diversity in mainland ‘*H. cf. duvaucelii*’ or the presence of at least one extinct, large, broad-toed diplodactylid species.

**Supplementary Information:**

The online version contains supplementary material available at 10.1186/s12862-021-01808-7.

## Background

New Zealand’s lizard fauna is characteristic of isolated archipelagos, exhibiting high species endemism, extensive in situ radiations [1, 2] and insular gigantism [3]. Despite this diversification, the New Zealand Diplodactylids (compared with the New Caledonian radiation) are relatively conserved in form; exhibiting reduced morphological divergence at a similar evolutionary depth [2, 4].

Formal taxonomic descriptions of New Zealand diplodactylid species [5, 6], similar to those of Australian [7, 8] and New Caledonian representatives [9–11], have been based exclusively on external morphological characters (e.g. coloration and scalation), with interspecific skeletal variation rarely analysed. Early anatomical studies [12], however, noted osteological differences between the two then recognized genera: *Hoplodactylus* (‘brown geckos’) and *Naultinus* (‘green geckos’); focussing on the neotenic condition of *Naultinus* and the ‘primitive’ osteology of the New Zealand Diplodactylidae. Despite these in-depth comparisons, re-examination of generic level skeletal variation in the context of current nomenclature is required, given considerable taxonomic fluidity over the last 65 years [13–15].

For example, allozyme [15, 16] and mitochondrial DNA [17, 18] analyses recognized three species complexes within the genus *Hoplodactylus*, corresponding to two broad morphological groupings: narrow-toed (*H. granulatus* and *H. pacificus*) and broad-toed (*H. maculatus*) clades [19]. Further taxonomic revision [2] separated multiple *Hoplodactylus* species complexes into five genera (*Dactylocnemis*, *Mokopirirakau*, *Toropuku*, *Tukutuku* and *Woodworthia*), with *Hoplodactylus* reserved for *H. duvaucelii* and the extinct giant *H. delcourti* [12]. Conversely, the monophyletic genus *Naultinus* has been historically over-split, separated into North and South Island genera (*Naultinus* and *Heteropholis* respectively; [13]), with *Heteropholis* later synonymised with *Naultinus* [19].

Descriptions of these revised genera were based exclusively on external morphology [2]; with osteological differences only recognized for the frontal [21, 22], which has led to assertions of skeletal uniformity at the species-level [23, 24]. In comparison, the identification of interspecific skeletal variation in cranial elements of New Zealand’s large eugongyline skinks has enabled both differentiation from smaller congeners [25], and description of the extinct ‘giant’ *Oligosoma northlandi* [26, 27] from Holocene subfossil remains. Classification of isolated diplodactylid remains however, has been restricted to size comparisons with extant representatives (in reference to now outdated taxonomy; [23]), particularly in the identification of ‘*H. cf. duvaucelii*’, New Zealand’s largest extant diplodactylid.

**Fig. 1:**
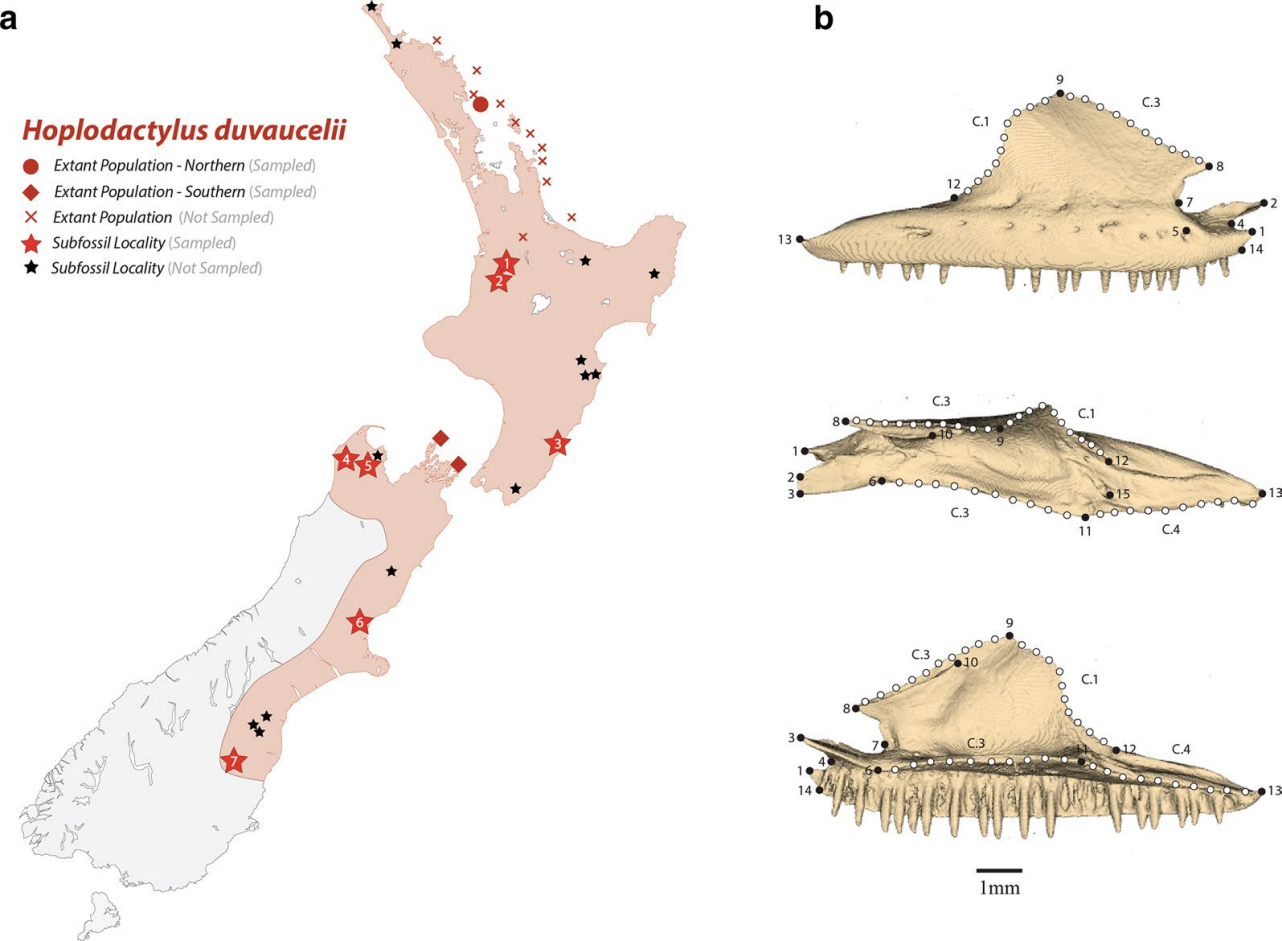
**a** Assumed Holocene distribution (red fill) of *Hoplodactylus duvaucelii*, showing extant modern northern/southern populations (circles, crosses and triangles) and subfossil collection localities (stars). Numbers denote sampled Holocene subfossil collection localities (1–7), with letters corresponding to subfossil specimens (A-J): Little Lost World, Waitomo (1—A); Companionway Cave, Waitomo (2—K); Mataikona River, Wairarapa (3—I); Gouland Downs, Tasman (4—G); Takaka Hill, Tasman (5—H); Ardenest, North Canterbury (6 – B/C/D/E/F); Earthquakes, North Otago (7—J). **b** Surface models of a diplodactylid maxilla in lateral (top), dorsal (middle) and medial (bottom) views demonstrating placement of fixed landmarks (black circles) and equally spaced semilandmarks (white circles). Numbers and C-prefixed numbers correspond to anatomical landmark descriptions (Additional file 1: Table S2.3)

*Hoplodactylus duvaucelii* (‘Duvaucel’s gecko’) is a large, nocturnal species, with a pseudoendemic (realized) distribution on predator-free islands in the Cook Strait and off the north-eastern coast of the North Island (Fig. 1A; [28]). Prior to Polynesian (~ 1280 AD; [29]) and European (effectively the late 1700s) arrival, ‘*H. cf. duvaucelii’* was widely distributed throughout the North Island [25], and the northwest and eastern South Island (Fig. 1a; [23, 30–33]), evidenced by the presence of large, isolated diplodactylid remains in Holocene predator (﻿laughing owl and falcon) middens. Subsequent range contractions occurred as a result of the synergistic effects of competitive exclusion, direct predation by introduced mammals, and degradation of forest habitat [25, 34]. This extensive distribution across multiple biogeographic regions [23, 35], given pronounced regional endemism recognised (or proposed) in other New Zealand diplodactylid genera [1, 2], combined with the extinction of other large lizards in New Zealand [26, 27] implies unrecognized diversity may exist within ‘*H. cf. duvaucelii*’; perhaps detectable through fine-scale osteological analysis. Geometric morphometrics [36], a method of statistical shape analysis that enables improved detection and visualisation of subtle morphological differences (compared with traditional linear-based morphometrics [37, 38]), has been widely applied in herpetofaunal studies; including the successful discrimination of closely-related species [39–41] and classification [42–44] of isolated cranial elements. We therefore predict osteological differences, if examined appropriately (using geometric morphometrics), will be sufficient for discriminating between extant diplodactylid genera (and potentially species), enabling the identification of isolated subfossil material, or reveal unidentified taxa that require description and diagnosis.

Herein, three-dimensional geometric morphometrics is used to characterise and quantify both shape and size variation in the maxilla of modern New Zealand diplodactylid genera (*Dactylocnemis*, *Hoplodactylus*, *Mokopirirakau*, *Naultinus* and *Woodworthia*), for comparison with Holocene ‘*H. cf. duvaucelii*’ subfossils. Three main research questions are tested: (a) can recognised diplodactylid genera be distinguished based on maxilla shape; (b) is size a reliable method for generic-level identification of isolated cranial elements; and (c) have cryptic extinctions occurred in the Diplodactylidae (with a focus on ‘*H. cf. duvaucelii*’)?

## Results

### Principal axes of diplodactylid maxilla variation

**Fig. 2:**
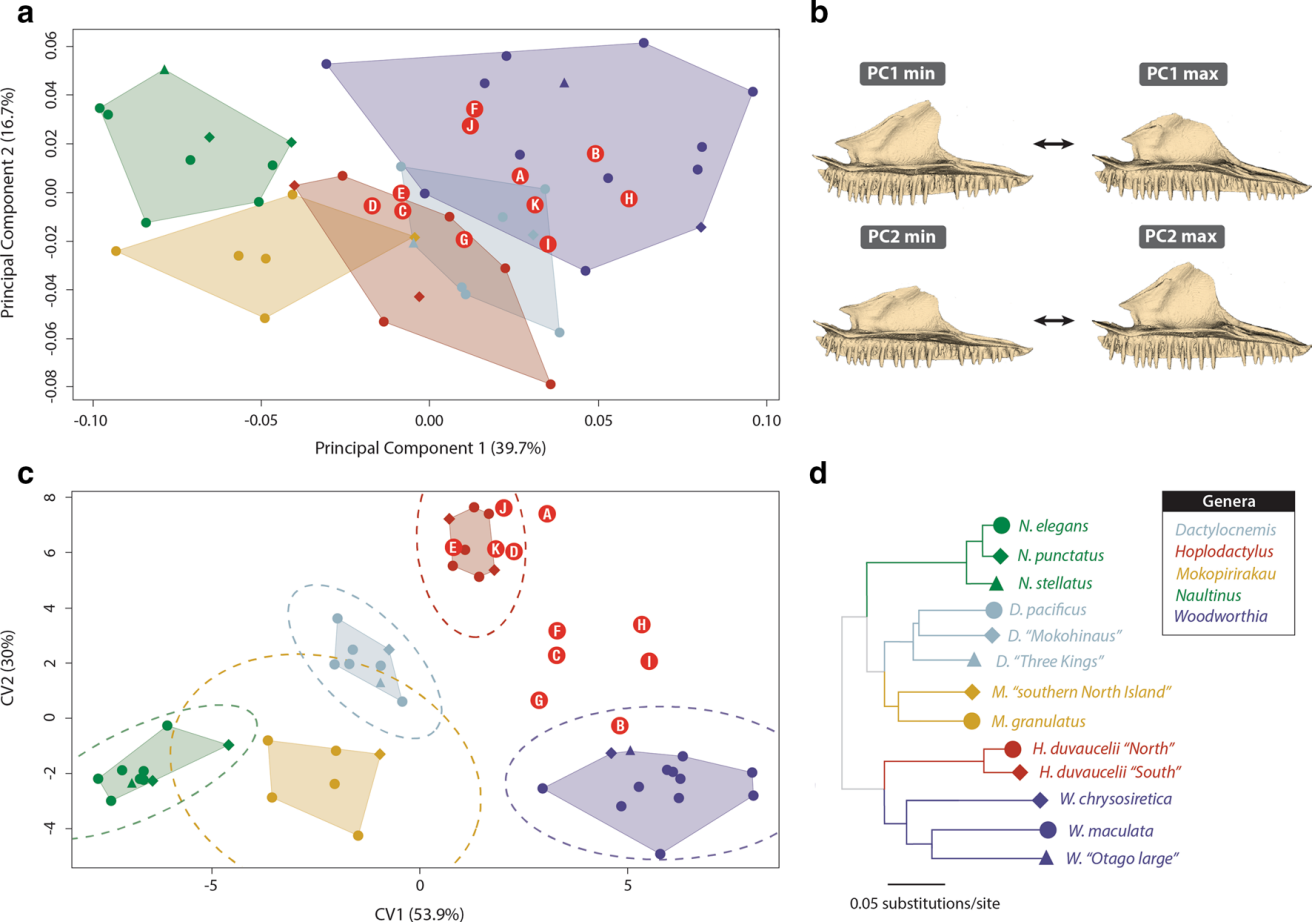
**a** Principal component (PC) analysis of maxilla shape showing PC1 versus PC2 (representing 56.4% of variation in maxilla shape). **b** Surface warps representing the maxima and minima shape differences of PC1/PC2 axes (see A). **c** Canonical variates (CV) analysis showing CV1 versus CV2 (representing 83.9% of the total among-group variance) with 95% confidence ellipses plotted for each genus. **d** Phylogenetic tree of described/undescribed diplodactylid species analysed (adapted from [2, 18]). Points in (A) and (C) are modern individuals (symmetric component of left–right maxilla shape) coloured by genus (*Dactylocnemis*: blue-grey, *Hoplodactylus*: red, *Mokopirirakau*: yellow, *Naultinus*: green, *Woodworthia*; purple) and bounded by convex hulls, with shapes (circle, diamond, triangle) corresponding to species shown in (D). Holocene subfossil individuals are shown as red circles (A-J): Waitomo (A: AU7700, K: WO333), Wairarapa (I: S.46528.1), Tasman (G: S.38813.2; H: S.39086), North Canterbury (B: S.33703.2, C: S.33703.3, D: S.33703.4, E: S.33703.7, F: S.33703.8) and North Otago (J: VT791a)

Principal component (PC) analysis of landmarked maxillae (Fig. 1b) reveals the majority (71.5%) of shape variability among extant New Zealand diplodactylids is concentrated in four dimensions (Fig. 2a; Additional file 1: Figure S3). Subsequent PC contributions (PC5—PC54) are either small or negligible (< 5.0%), and thus not considered further.

PC1, the primary axis of maxilla shape variation (39.7%), largely pertains to differences in shape of the nasal and orbital margins (Fig. 2b; Additional file 1: Figure S4). Negative PC1 values are associated with an elongate nasal margin and adjacent medial flange; more concave prefrontal and orbital margins; and a more convex palatal shelf. *Naultinus* and *Mokopirirakau granulatus* (i.e. excluding the *M.* ‘southern North Island’ specimen—see below) form distinct clusters in the negative region of PC1, whereas *Dactylocnemis*, *Hoplodactylus* and *Woodworthia* primarily occupy overlapping intermediate regions of the positive zone (Fig. 2a).

PC2 (16.7% of variance) describes differences associated with overall element robustness, with dorsoventrally shallow, laterally slender maxillae along the negative sector of the axis, contrasting with dorsoventrally deep, laterally broad maxillae along the positive region of the axis (Fig. 2b; Additional file 1: Figure S4). Two morphologically distinct generic clusters form along this axis: robust maxillae (*Naultinus-Woodworthia*) and gracile maxillae (*Dactylocnemis-Hoplodactylus-Mokopirirakau*; Fig. 2a).

PC3 (8.0% of variance) describes shape differences in both the anterolateral lappet and prefrontal margin (Additional file 1: Figure S4); with dorsoventral thickening (and lateral thinning) of the anterolateral lappet and plateauing of the prefrontal margin at negative values. Conversely, at positive values, the prefrontal margin forms a near continuous curve with the adjacent orbital margin (Additional file 1: Figure S4). Shape differences along PC4 (7.2%) are primarily associated with increased curvature of the tooth row along the negative sector of the axis (Additional file 1: Figure S4).

Visually, Holocene subfossil specimens cluster in the intermediate region of the positive zones of PC1, PC2 and PC4; overlapping the morphospaces of extant genera (Fig. 2a; Additional file 1: Figure S3). Conversely, Holocene subfossil specimens (excluding H) occupy increasingly positive regions of PC3, with some individuals (B, E, I, J) exhibiting no overlap with extant genera (Additional file 1: Figure S3). Procrustes distances of the Holocene subfossil specimens (Additional file 1: Table S4) across all PC axes suggest shape similarities with *Dactylocnemis* (E, K), *Hoplodactylus* (A, C, D, G, I, J) and *Woodworthia* (B, F, H), with no shape similarities with *Mokopirirakau* or *Naultinus.*

### Predictors of shape and size

Procrustes ANOVA (Additional file 1: Table S5) revealed that phylogenetic affiliation (i.e. genus) is a highly significant predictor (F_(4,38)_ = 9.01, *p* < 0.001) of maxilla shape, accounting for 45.2% of the shape variation. Multivariate pairwise *post-hoc* tests indicate differences to be significant between most genera (*p* < 0.05), excluding *Dactylocnemis-Hoplodactylus* (*p* = 0.229), and *Hoplodactylus-Mokopirirakau* (*p* = 0.056) comparisons (Additional file 1: Table S6). A weak but significant relationship also exists between maxilla shape and centroid size (F_(1,41)_ = 5.39,* p* = 0.020), and their interaction (F_(4,38)_ = 1.35, *p* = 0.023; Additional file 1: Table S5), suggesting a small proportion of the shape diversity (6.8%) is due to allometry.

One-way ANOVA (Additional file 1: Table S7) identified significant differences in maxilla centroid size between genera (F_(4,38)_ = 32.22, *p* < 0.001), with *Hoplodactylus* (1690 ± 228.1; mean ± sd) being significantly larger under all HSD *post-hoc* comparisons (Additional file 1: Table S8). Additionally, *Woodworthia* (968 ± 100.9) is significantly smaller than most other genera (Additional file 1: Figure S5; Table S8), excluding the *Naultinus-Woodworthia* comparison (*p* = 0.253). Conversely, *Dactylocnemis* (1198 ± 142.9), *Mokopirirakau* (1241 ± 115.6) and *Naultinus* (1093 ± 104.0) are indistinguishable from each other based on centroid size alone. Subfossil specimens show no overlap with the error bars of non-*Hoplodactylus* maxillae, with some (G = 2042, H = 2086, I = 2024, K = 2316) extending beyond the maximum extant *Hoplodactylus* maxilla centroid size (Additional file 1: Figure S5).

### Phylogenetic shape differences

Phylogenetic signal in maxilla shape is statistically significant (K_mult_ = 0.828, *p* < 0.001); however, species resemble each other less than expected under a model of Brownian motion (given K_mult_ is less than 1). This is reflected in the distribution of species throughout the phylomorphospace (Additional file 1: Figure S6), with several overlapping branches (e.g. *Dactylocnemis* and *Hoplodactylus*) and non-adjacent closely related species (e.g. *M. granulatus* and *M.* ‘southern North Island’).

Canonical variate (CV) analysis (Fig. 2c) and Mahalanobis distance probabilities (Additional file 1: Table S9) show all genera form significantly different groups, with a cross-validation accuracy of 100%. Canonical function 1 (CV1; 53.9% among-group variance) clearly distinguishes *Naultinus* and *Woodworthia*, which occupy opposite extremes of the morphospace (Fig. 2c). The positive sector of CV1 describes shortening of the nasal margin and adjacent medial flange, with corresponding shortening in the prefrontal margin (similar to PC1; Additional file 1: Figure S7). *Hoplodactylus* occupies the extreme positive region of canonical function 2 (CV2; 30% among-group variance), characterized by a relative slope decrease of the nasal margin and consequent shortening of the orbital margin (Fig. 2c; Additional file 1: Figure S7).

The Holocene subfossil specimens are broadly distributed throughout the positive zones of CV1 and CV2 (Fig. 2c), with some individuals visually falling within the 95% confidence-interval of the morphospaces of extant genera (*Hoplodactylus*: D, E, J, K; *Woodworthia*: B). Typicality probabilities of Mahalanobis distances across all CVs (Table 1) show that while many Holocene subfossil specimens strongly associate with *Hoplodactylus* (A, D, E, F, J, K), other specimens (B, C, G, H, I) show no clear phylogenetic affinities, indicating that Holocene subfossil specimens display greater variation in maxillary form than that encompassed by the extant genera. Conversely, despite posterior probabilities (Table 1) showing similar significant support for Holocene subfossil *Hoplodactylus* classification (A, C, D, E, F, H, J, K), specimens with lower typicality probabilities were assigned to *Woodworthia* (B, G, I).

**Table 1:** Typicality and posterior probabilities of Holocene subfossil specimens belonging to extant genera, calculated using Mahalanobis distances

		Typicality probabilities					Posterior probabilities				
		D	H	M	N	W	D	H	M	N	W
A	AU7700	0.026	**0.215**	0.020	0.018	0.033	< 0.001	**1**	< 0.001	< 0.001	< 0.001
B	S.33703.2	0.013	0.030	0.021	0.013	0.041	< 0.001	< 0.001	< 0.001	< 0.001	**0.999**
C	S.33703.3	0.031	0.110	0.046	0.026	0.070	< 0.001	**0.999**	< 0.001	< 0.001	< 0.001
D	S.33703.4	0.257	**0.688**	0.096	0.046	0.122	< 0.001	**1**	< 0.001	< 0.001	< 0.001
E	S.33703.7	0.570	**0.819**	0.133	0.072	0.120	< 0.001	**0.999**	< 0.001	< 0.001	< 0.001
F	S.33703.8	0.157	**0.557**	0.125	0.060	0.286	< 0.001	**0.999**	< 0.001	< 0.001	< 0.001
G	S.38813.2	0.031	0.060	0.054	0.024	0.061	< 0.001	0.019	0.004	< 0.001	**0.809**
H	S.39086	0.041	0.167	0.043	0.020	0.114	< 0.001	**0.999**	< 0.001	< 0.001	< 0.001
I	S.46528.1	0.028	0.091	0.044	0.016	0.090	< 0.001	0.049	< 0.001	< 0.001	**0.512**
J	VT791a	0.078	**0.205**	0.027	0.022	0.038	< 0.001	**1**	< 0.001	< 0.001	< 0.001
K	WO333	0.177	**0.742**	0.088	0.050	0.105	< 0.001	**1**	< 0.001	< 0.001	< 0.001

Highest typicality (*p* > *0.20*) and posterior probabilities for each Holocene subfossil specimen are indicated in bold

## Discussion

### Variation and morphological convergence in diplodactylid maxillae

Phylogenetic position is a significant predictor of maxilla shape diversity in New Zealand diplodactylids, with all genera (*Dactylocnemis*, *Hoplodactylus*, *Mokopirirakau*, *Naultinus* and *Woodworthia*) being morphologically distinct. Identification of taxonomically informative morphological variation within a single skeletal element contrasts with previous assertions of skeletal uniformity in New Zealand’s geckos (e.g. [23, 26]).

Variation in diplodactylid maxilla shape is predominantly explained by two characters, described by the first two axes of both PCA and CVA: (a) posterior extension/reduction of the nasal margin; and (b) increase/decrease in dorsoventral extent of the facial process. Separation of genera along PC1 appears correlated with broad habitat use of New Zealand diplodactylids, with terrestrial-arboreal (*Dactylocnemis*, *Hoplodactylus* and *Woodworthia*) and exclusively arboreal (*Naultinus*) genera occupying positive and negative regions respectively [45, 46]. This morphological signature of habitat use extends to species-level comparison, most notably for discrimination of the terrestrial-arboreal *M.* ‘southern North Island’ from the arboreal *M. granulatus* [47], characterized by a shift to more positive values.

In lizards, arboreal forms tend towards broad, pointed skulls, and, similar to saxicoline species, tend to be dorsoventrally flattened, presumably enabling faster climbing speeds on non-horizontal surfaces [48–50]. While cranial modifications associated with habitat use are undocumented for New Zealand diplodactylids, extension of the nasal margin in arboreal species appears to be linked to two superficial morphological changes in the adjacent prefrontal margin: (a) a reduction in anterior extent (observed in other Gekkota; [51]); and (b) formation of a thickened ridge along the prefrontal orbital margin (Additional file 1: Figure S8). While the function of these features remains unclear, association with arboreality may indicate ecomorphological convergence between phylogenetically independent lineages. Despite describing similar shape change, separation of genera along CV1 reflects broad phylogenetic relationships, distinguishing broad (*Hoplodactylus*, *Woodworthia*) and narrow (*Dactylocnemi*s, *Mokopirirakau*, *Naultinus*) toed clades by positive and negative values respectively; supporting previous morphological classification [16].

In addition to habitat use, skull-shape evolution in lizards is strongly influenced by diet, with shape variation concentrated in the premaxilla, nasal and jaw joint, reflecting their roles in jaw-based prehension and feeding biomechanics [48, 52]. Herbivorous lizard skulls tend towards reduced snout lengths and high temporal regions relative to carnivorous lizards, contributing to increased bite strength required for processing fibrous and tough foliage [53–55]. Conversely, omnivorous gekkotans represent intermediate forms not specialized for particular feeding behaviors, and consequently lack unique morphological adaptations [56]. New Zealand geckos are predominantly omnivorous, consuming a wide variety of food items, including plant matter (fruit, honeydew and nectar) and arthropods [46]. Such extensive dietary overlap affects the use of diet as a variable of maxilla shape, given that the categories (omnivorous and insectivorous) are not discrete.

Finally, as lizard maxillae are evolutionarily conserved, exhibiting reduced disparity relative to rate of evolution (compared with other cranial elements; [52]); similar analyses of elements critical to cranial biomechanics (e.g. quadrate, which also supports the auditory system) may enhance detection of stronger species-level morphological signals in the New Zealand Diplodactylidae.

### Efficacy of size-based discrimination

Maxilla size is significantly correlated with phylogenetic affinity; however, only *Hoplodactylus* can be fully differentiated (under *post-hoc* comparisons), with the remaining diplodactylid genera exhibiting variable degrees of overlap. This highlights the inefficiency of previous size-based taxonomic identification of non-*Hoplodactylus* Holocene subfossil geckos, especially intermediate-sized genera (*Dactylocnemis*, *Mokopirirakau* and *Naultinus*), which exhibit complete size overlap. Similarly, while large size proves reliable in discriminating extant *H. duvaucelii*, application to Holocene subfossil identification requires assumptions of temporal taxonomic homogeneity (or “covert biases”; [57]).

Previous analyses of squamate genera including *Anolis* [58, 59] and *Iguana* [60] have shown maxillae to be effective predictors of snout-vent length (SVL). Our results exhibit similar trends both between and within diplodactylid genera, with mean genus centroid size reflecting relative SVL [61], and larger species (*N. punctatus*, *D.* ‘Three Kings’) having increased centroid sizes relative to smaller congeners (*N. elegans*, *D. pacificus*; [62, 63]).

### Increased Holocene diversity of large geckos

Our results provide evidence for increased morphological diversity of large geckos during the Holocene in New Zealand, with declines in both shape and size variation following Polynesian and European colonization. This suggests that well-characterized biodiversity reductions (and extinctions) observed across insular avifauna [64, 65] extend to lineages comprised of taxa of smaller body size, including herpetofauna.

Combined Procrustes and Mahalanobis distance comparisons provide support for previous size-based assignment of five Holocene subfossils (A, D, E, J, K) to *H. duvaucelii*, confirming assumed prehuman distribution of this species across both the North and South Islands. The remaining six Holocene subfossil specimens (B, C, F, G, H, I) exhibit classificatory discrepancies and/or reduced assignment probabilities (below relevant thresholds), reflected by their unique position across CV1/CV2. These distinct Holocene subfossil maxillae (“unknown taxa”) do not reflect differential adaptation of *H. duvaucelii* to mainland and island habitats (given shape overlap of mainland and island populations) but reflect either increased morphological diversity of mainland large species (not encompassed by extant populations) or the presence of at least one extinct, large, broad-toed diplodactylid species.

Based on digit morphology, the extinct giant *H. delcourti* was positioned within the broad-toed clade, sister to *H. duvaucelii* [20], suggesting these “unknown taxa” could potentially represent small or even juvenile *H. delcourti*. However, this seems unlikely given the paucity of reported subfossil remains of *H. delcourti* [66], despite extensive collections of other diplodactylid taxa [26]. More precise phylogenetic affinities of both *H. delcourti* and “unknown taxa” could be determined through future ancient DNA analysis.

During the Holocene, mainland *H. duvaucelii* (and “unknown taxa”) reached larger sizes than extant populations, reflected in a reduction in maximum maxilla size (a proxy for body size; e.g. [58]). Such sized-biased extinction is well-documented for Quaternary insular lizards globally [59, 67–69], including the extinction of two large-bodied eugongyline skink species (*Oligosoma northlandi* and *Oligosoma sp.*) in northern New Zealand [25, 27, 70]. This reflects the inherent vulnerability of New Zealand’s large-bodied, nocturnal herpetofauna to high predation rates and ecological displacement by exotic mammals (including the Pacific rat *Rattus exulans* (kiore); [71, 72]), particularly in forest-cleared environments [73]. Smaller lizards can escape predation during periods of inactivity through utilizing narrow retreats, given limited overlap in body diameter with small mammalian predators [45]. Conversely, refugia utilized by large-bodied lizards can be accessed by mammalian predators, as evidenced by reductions in body weight, tail width and recruitment of *H. duvaucelii* on kiore-inhabited islands [34, 74].

Similarly to extant *H. duvaucelii* populations [75], Holocene subfossil *H. duvaucelii* also exhibit a latitudinal cline in maxilla size, in opposition to Bergmann’s rule (i.e. increased size at higher latitudes), with individuals from northern localities being noticeably larger than those from southern localities. For diurnal lizards, reduced body size appears to be an advantageous thermoregulatory strategy in cooler climates, with high surface-area to volume ratio permitting rapid heat gain whilst sun-basking [76, 77]. Despite being nocturnal, Duvaucel’s gecko occasionally emerges from retreats to thermoregulate through cryptic sun-basking [78, 79], suggesting that small body size provides an adaptive advantage at high latitudes.

## Conclusions

The majority of New Zealand diplodactylid genera can be differentiated from each other based exclusively upon the shape of the maxilla, which exhibits strong correlations with phylogenetic relationships. Additional species-level discrimination based on ecomorphological adaptations highlights the potential application of geometric morphometrics to the morphological characterization of highly functionally variable elements (or whole skulls) in taxonomic descriptions of extant diplodactylid species. Previous sized-based identification of Holocene subfossils is ineffective and underestimates extinct diversity, suggesting global assemblages of insular reptiles are depauperate in comparison to their prehuman diversity.

## Methods

### Specimen selection

To capture extant morphological variation, we examined both left and right maxillae (sensu [80]) from 43 adult skeletal specimens (Additional file 1: Table S1) representing 13 species from five diplodactylid genera: *Dactylocnemis, Hoplodactylus, Mokopirirakau, Naultinus* and *Woodworthia* (Additional file 1: Figure S1, Table S1). In addition, we examined 11 well-preserved Holocene subfossil maxillae identified as ‘*Hoplodactylus cf. duvaucelii*’, covering the majority of their (assumed) prehuman range (Fig. 1a; Additional file 1: Table S1). Maxillae were utilized primarily due to their relative abundance in subfossil deposits (Scarsbrook pers. obs.), compared with more osteologically informative elements (e.g. quadrate; [81, 82]). For additional specimen selection details see Additional file 1: Methods.

### Geometric morphometrics

Geometric morphometric analyses were performed on a total of 94 maxillae (see Additional file 1: Methods for additional analytical details). Three-dimensional rendered surface models were generated from micro-CT reconstructions of maxillae, with shape characterized by 15 landmarks and 40 sliding semi-landmarks (Fig. 1b; Additional file 1: Figures S2, Table S2) digitized in Checkpoint (Stratovan Corporation, Davis, CA). Landmark coordinates were aligned using a generalized least-squares Procrustes superimposition [38], with semi-landmark position optimized using the Procrustes distance criterion [83] and paired elements symmetrized (following mirroring of left maxillae coordinates; Additional file 1: Table S3).

Shape variation in maxillae of the extant species was assessed using principal component analysis (PCA); with intergeneric differences (shape ~ genus * size) tested using a Procrustes analysis of variance (ANOVA; [84]), and visualized using canonical variate analysis (CVA; [85]) with cross-validations, based on a reduced set of PC scores [86, 87]. Three-dimensional surface warps [88] representing minimum and maximum shapes along both principal component (PC) and canonical variate (CV) axes were generated using the thin-plate spline (TPS) method [87, 89]. Phylogenetic signal in maxilla shape was calculated using K_mult_ [90, 91], with statistical significance determined using phylogenetic permutation (tree inferred from [2, 18]; Fig. 2d) with 1000 iterations [92]. Interspecific phylogeny-associated shape variation was visualized across the first two PC axes [92].

Holocene subfossil maxillae were then projected into these two-dimensional morphospaces (i.e. PCA and CVA) through matrix multiplication with respective eigenvectors (e.g. [93]). Phylogenetic classification of Holocene subfossil specimens was performed through Procrustes and Mahalanobis distance comparisons (to the mean maxilla shape of each genus), with the latter used to calculate typicality [94, 95] and posterior [96] probabilities Variation in size of the maxilla (represented as centroid-size of the landmark configuration) between genera was examined using a one-way ANOVA and Tukey's honestly significant difference (HSD) *post-hoc* tests [97], and visualised using a barplot. All statistical analyses were performed in the R statistical environment v. 3.6.1 [98] using the packages *geomorph* v. 3.1.2 [92] and *Morpho* v. 2.7 [99].

## Supplementary Information

**Additional file 1.** Supplementary methods, figures (S1-S8) and tables (S1-S8).

**Additional file 2.** Three-dimensional landmark coordinates for modern and subfossil maxilla.

**Additional file 3.** R code for geometric morphometric and statistical analyses.

## Data Availability

The dataset (i.e. raw landmark coordinates and R-code) supporting the conclusions of this article is included within the article and its Additional file 2 and Additional file 3.
