# Supplementary material for: Skeletal variation in extant species enables systematic identification of New Zealand’s large, subfossil diplodactylids
Source: BMC Ecol Evol. 2021 Apr 27;21:67. doi: 10.1186/s12862-021-01808-7 (PMC8080345; doi:10.1186/s12862-021-01808-7)
Supplement: Supplementary file 1 — Additional file 1. Supplementary methods, figures (S1-S8) and tables (S1-S8). [file 12862_2021_1808_MOESM1_ESM.pdf]

Supplementary Material for:

Skeletal variation in extant species enables systematic identification of New Zealand's large, subfossil diplodactylids.

Lachie Scarsbrook[1], Emma Sherratt[2], Rodney A. Hitchmough[3], Nicolas J. Rawlence[1]

[1] Otago Paleogenetics Laboratory, Department of Zoology, University of Otago, Dunedin, New Zealand
[2] School of Biological Sciences, The University of Adelaide, Adelaide, South Australia, Australia
[3] Department of Conservation, Wellington, New Zealand

This PDF file includes:

Supplementary Methods
Supplementary Figures (S1-S8)
Supplementary Tables (S1-S8)
References for Supplementary Material

Other supplementary materials for this manuscript include the following:

Dataset 1. Raw Landmark Coordinates
RCode. GMM Analysis

Table of Contents

Supplementary Methods **2**
(a) Study specimens 2
(b) Imaging 3
(c) Landmark acquisition and morphometric analysis 3
(d) Principal component analysis 4
(e) Comparative statistical analysis 4
(f) Phylomorphospace 5
(g) Canonical variate analysis 5
Supplementary Figures (S1-S8) **7**
Supplementary Tables (S1-S8) **15**
References for Supplementary Material **25**

Supplementary Methods

(a) Study specimens

To capture extant morphological variation, we examined both left and right maxilla (*sensu* (1)) from 43 adult skeletal specimens (Supplementary Table 1) representing 13 species from five diplodactylid genera: *Dactylocnemis, Hoplodactylus, Mokopirirakau, Naultinus* and *Woodworthia* (Supplementary Figure 1). Maxillae were utilized as they were known to be taxonomically informative in fossil lizards (2,3) and relatively abundant in subfossil collections given preservation (paired structure) and identification (characteristic Gekkonidae morphology; (4)) biases. Samples were chosen based on availability in museum collections, resulting in limited sample sizes and absence of rarer genera, namely *Toropuku* and *Tukutuku* (e.g. (5)). Species-level determination was based on collection locality (Hitchmough, pers. comm. 2020) given classification prior to significant taxonomic revisions (6). Accordingly, most statistical analyses were restricted to inter-genera, with differences between species visualized through species specific notation on plots. Additionally, northern and southern Duvaucel's gecko (*Hoplodactylus duvaucelii*) populations were distinguished with similar notations, given extensive genetic distance (e.g. (7)).

Despite being observed in other squamates (8–10), morphological diversity attributable to sexual dimorphism was not examined, as sex was not recorded for the majority (63%) of specimens. To determine how maxilla shape influences juxtaposing cranial elements (prefrontal, frontal and nasal), whole-skull micro-computed tomography (micro-CT) scans of a representative individual from each genus analysed were downloaded (with author permission; Paluh et al. 2018) from MorphoSource (Identifiers: S15380; S15404; S15417; S15420; S15463; https://www.morphosource.org/). In addition, we examined 11 well-preserved, Holocene subfossil maxillae identified as '*Hoplodactylus cf. duvaucelii*' (Supplementary Table 1), covering the majority of their assumed prehuman range (Figure 1). Lack of sampling from known Holocene subfossil localities (e.g. Figure 1) reflected either an absence of maxillae (in museum collections), or poor morphological preservation of recovered elements. Maxilla morphology (Supplementary Figure 2) was characterized following Evans (11), Gray et al. (2) and Ledesma and Scarpeta (12).

(b) Imaging

3D rendered surface models were generated from X-ray micro-Computed Tomography (micro-CT) reconstructions of both extant and Holocene subfossil maxilla. Specimens were micro-CT scanned at a resolution of ~16 μm (typically 50 kV, 200 μA), using a Skyscan 1172 (Bruker micro-CT) at the Otago Micro and Nanoscale Imaging facility (OMNI). Scattering artefacts were reduced using a 0.5 mm aluminium filter. The raw X-ray images (shadowgrams) were reconstructed into volumes using the NRecon software interface (Skyscan, Aartselaar, BE) and sliced transversely, producing image stacks (.TIFF), from which individual maxilla were digitally isolated using the grayscale threshold method in FIJI v. 2.0.0 (13).

(c) Landmark acquisition and morphometric analysis

Maxilla shape was characterized using 3D Cartesian coordinates of 15 fixed landmarks (representing equivalent anatomical loci) and 40 equally spaced semi-landmarks (demarcating four major homologous curves; Figure 1B; Supplementary Table 2), manually digitized using Checkpoint v. 2019.03.04.1102 (Stratovan Corporation, Davis, CA). Landmark digitization was duplicated, and the specimen order randomized to minimize measurement and systematic errors respectively (14). Coordinate data were exported as individual Morphologika files, with reflected copies of each left maxilla generated through reversing the sign of the x-coordinates (for symmetric analysis below). All subsequent statistical analyses were performed in the R statistical environment v. 3.6.1 (15) using the packages *geomorph* v. 3.1.2 (16) and *Morpho* v. 2.7 (17).

The 3D landmark coordinates for extant maxillae (n = 83) were aligned using a generalized least-squares Procrustes superimposition (effectively removing differences in size, position and orientation; (18)), taking into account both matching symmetry and replicate, resulting in shape variables for the symmetric component of shape (19). To minimize Procrustes distance between specimens, semi-landmarks were permitted to slide along their tangent directions during Procrustes superimposition (20). As snout-vent length measurements were not available for most skeletal specimens, centroid size (calculated as the square root of the sum of squared distances of each landmark prior to Procrustes superimposition) was used as a proxy for body size (21). Procrustes mean square estimates for individual variation and fluctuating asymmetry (of both shape and size) exceeded measurement error under initial Procrustes ANOVA,

suggesting effects of measurement error are negligible ((19); Supplementary Table 3). The resulting symmetric shape data (averaged left and right shape configurations; (19)) were used in all subsequent analyses.

(d) Principal component analysis

Principal component analysis (PCA) was performed on the extant dataset to visualize maxilla shape variation among genera. Holocene subfossil specimens were later projected into this morphospace through matrix multiplication with the PCA eigenvectors (e.g. (22)). To interpret shape differences described by the major axes of shape variation identified by the PCA, a two-dimensional morphospace was plotted with points identified by species. To visualize shape differences throughout the PCA morphospace, 3D surface warps (23) representing shape change along principal component (PC) axes were generated using the thin-plate spline (TPS) method (24). Specifically, a triangular surface mesh closely resembling the mean shape (*Hoplodactylus cf. duvaucelii*, S.33703.3) was warped into the mean configuration of all symmetric shape coordinates using TPS (e.g. (25)). This reference mesh was subsequently warped into the shapes represented by the minima and maxima of the first four PC axes, in addition to the mean shape of each genus. Additionally, Procrustes distances for inter-genera and genera-subfossil comparisons were calculated as Euclidean distances in tangent space (21).

(e) Comparative statistical analysis

Procrustes analysis of variance (ANOVA) was performed on the extant dataset (using the '*procD.lm*' function; (26)) to determine whether genera occupy different regions of the morphospace (thus exhibiting distinct maxilla morphologies), in addition to testing the effect of size on maxilla shape. Statistical significance of shape differences ($p < 0.05$) was assessed using Goodall's F-ratio (27) and a randomized residual permutation procedure using 10,000 iterations (28,29).

A Bartlett's test of homogeneity of variances (between genera) was performed on centroid size data (Bartlett's $K^2 = 7.232$, $p = 0.124$) to ensure assumptions of ANOVA were satisfied. Once validated, one-way ANOVA was used to examine differences in centroid size between genera, with statistical significance ($p < 0.05$) assessed using Tukey's honestly significant difference (HSD) *post-hoc* test (30).

(f) Phylomorphospace

Phylogenetic signal in maxilla shape was calculated using K_{mult} (31), a generalization of Blomberg's K-statistic appropriate for high-dimensional and multivariate data (32). Under a Brownian motion model of trait evolution, K_{mult} has an expected value of 1.0, with higher K_{mult} values indicating increasing phylogenetic signal, thus greater morphological variance between, rather than within clades (32). Statistical significance of K_{mult} was determined using phylogenetic permutation with 1,000 iterations; calculated by permuting the mean symmetric shape data of each extant species among all tips of the phylogenetic tree (using the '*physignal*' function; (16)) inferred from (33) and (6). Phylogeny-associated shape variation (between species) was visualized using the '*phylomorphospace*' function (16) across the first two PC axes.

(g) Canonical variate analysis

Canonical variate analysis (CVA) with cross-validations (for dataset calibration) was used to reveal the morphological shape variables that maximize intergeneric variance relative to intrageneric variance, and to predict the potential phylogenetic position of Holocene subfossil specimens (22,34). CVA was performed on a reduced set of PC scores (representing 95% of the cumulative variation) to ensure the dimensionality of shape variables (n = 19) was less than the number of specimens (n = 43), in addition to removing minor components of non-shape (i.e. measurement error) variation (35,36). 95% confidence intervals were generated around each of the extant genera within the CVA morphospace. Holocene subfossil specimens were then projected into this morphospace using the canonical variates. As CVA ordinations do not preserve Procrustes geometry, Mahalanobis distances were used in subsequent analyses to correct for shape-space distortions from Euclidean space (36).

Typicality and posterior probabilities concerning Holocene subfossil phylogenetic classification were calculated using generalized distances (D): the Mahalanobis distance of Holocene subfossil specimens to the mean of each genus, adjusted by the standard deviation (37). The squared distance (D^2) was used to calculate chi-square (typicality) probabilities with p (number of discriminating variables) degrees of freedom, assuming multivariate normal within-group distribution (38). Typicality (or 'unrestricted') probabilities are a multivariate extension of the univariate t-test, which evaluates whether a single observation belongs to a

group, enabling Holocene subfossils to be classified as outliers ($p < 0.20$) with respect to the extant dataset (39–41). Conversely, posterior (or 'restricted') probabilities, which require additional standardization through comparison to intrageneric distances (resampled 10,000 times), force Holocene subfossil specimens to belong to an existing genus (38). If the distance between a Holocene subfossil specimen and a genus mean was greater than 95% ($p = 0.05$) of the within-genera differences, the null hypothesis: that the Holocene subfossil belongs to that genus, was rejected (e.g. (22)). Partial warps, representing the maximum and minimum shape along canonical variate (CV) axes, were generated by regression analysis of CV scores against maxilla shape variation and visualized using TPS (36).

Supplementary Figures (S1-S8)

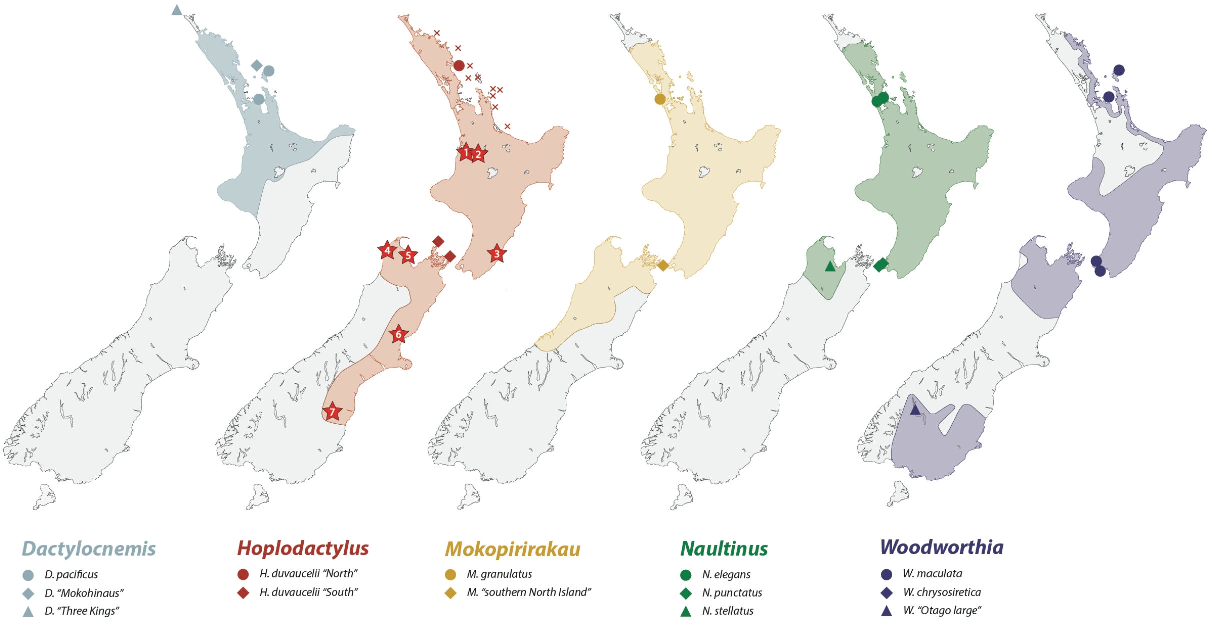

Supplementary Figure 1 Collection localities of diplodactylid specimens (circles, diamonds and triangles), with concatenated species distributions shown for each genera [39]. For *Hoplodactylus duvaucelii*, extant pseudoendemic island populations (crosses) and subfossil collection localities (numbered stars) are shown, with the illustrated range reflecting prehuman distribution (assumed). Numbers denote Holocene subfossil collection localities (1-7), with letters corresponding to subfossil specimens (A-J): Little Lost World, Waitomo (1 - A); Companionway Cave, Waitomo (2 - K); Mataikona River, Wairarapa (3 - I); Gouland Downs, Tasman (4 - G); Takaka Hill, Tasman (5 - H); Ardenest, North Canterbury (6 – B/C/D/E/F); Earthquakes, North Otago (7 - J).

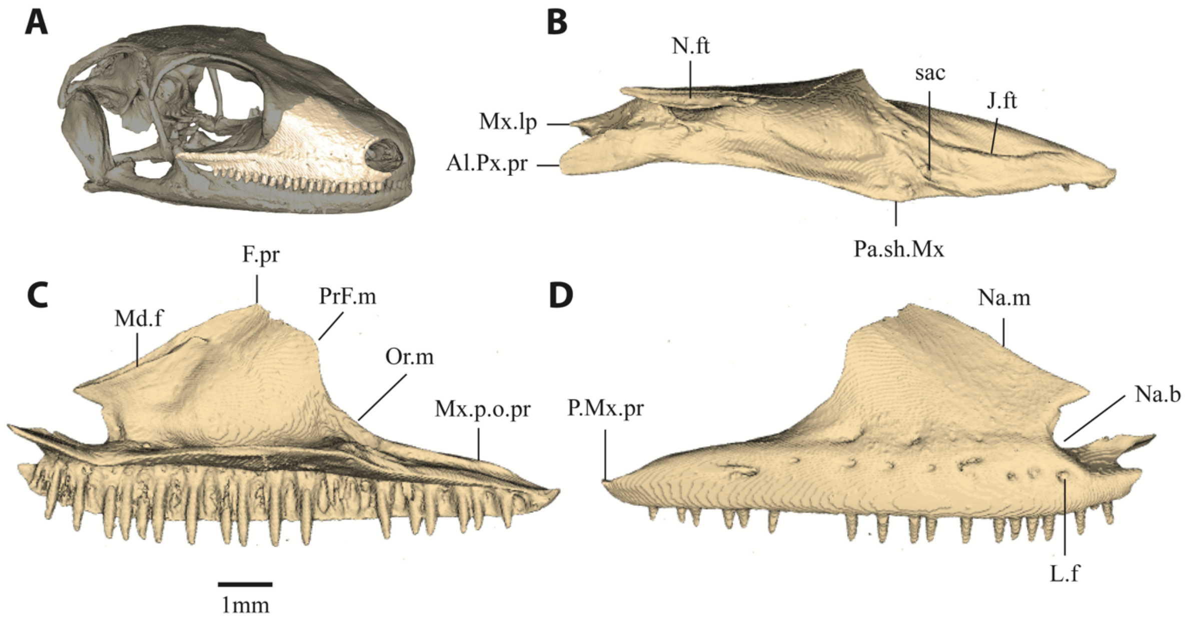

Abbreviation	Anatomical Feature	Abbreviation	Anatomical Feature
A.pr	Medial process	N.ft	Nasal facet
Al.Px.pr	Anterolateral lappet of premaxillary process	Na.b	Nasal basin
F.Mx5	Foramen/foramina for maxillary division of CN5	Na.m	Nasal Margin
F.pr	Facial process of maxilla	Or.m	Orbital Margin
J.ft	Jugal facet	P.Mx.l	Posterior maxillary lamina
L.f	Lateral foramen	P.Mx.pr	Posterior maxillary process
Md.f	Medial flange	Pa.sh.Mx	Palatal shelf of maxilla
Mx.lp	Maxillary lappet	PrF.m	Prefrontal margin
Mx.p.o.pr	Posterior orbital process of maxilla	sac	Superior alveolar canal

Supplementary Figure 2 Surface model of a representative diplodactylid skull (A) highlighting position of maxilla; and maxilla shown in anterior (B), medial (C) and lateral (D) views highlighting anatomical features associated with landmarks (abbreviations explained in the table).

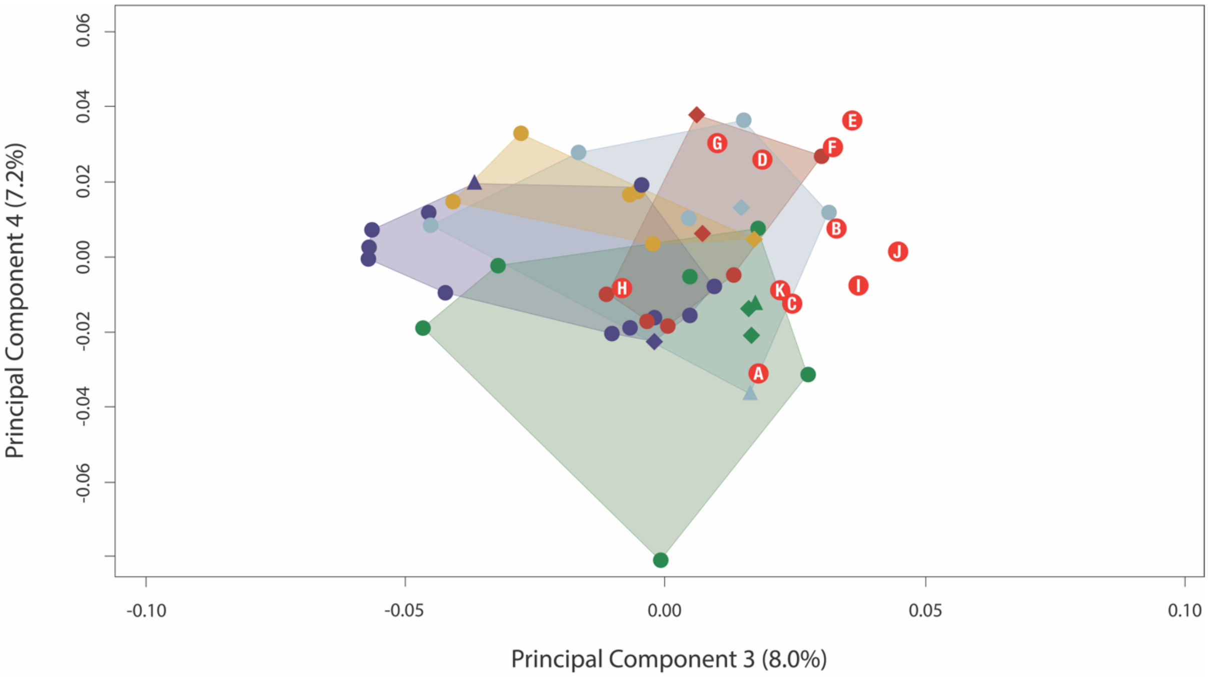

Supplementary Figure 3 Principal component (PC) analysis of maxilla shape showing PC3 versus PC4 (which represent 15.2% of variation in maxilla shape). Points are modern individuals (symmetric component of left-right maxilla shape) coloured by genus (*Dactylocnemis*: blue-grey, *Hoplodactylus*: red, *Mokopirirakau*: yellow, *Naultinus*: green, *Woodworthia*; purple) and bounded by convex hulls, with shapes (circle, diamond, triangle) corresponding to species (shown in Figure 1B). Holocene subfossil individuals are shown as red circles (A-J): Waitomo (A: AU7700, K: WO333), Wairarapa (I: S.46528.1), Tasman (G: S.38813.2; H: S.39086), North Canterbury (B: S.33703.2, C: S.33703.3, D: S.33703.4, E: S.33703.7, F: S.33703.8) and North Otago (J: VT791a).

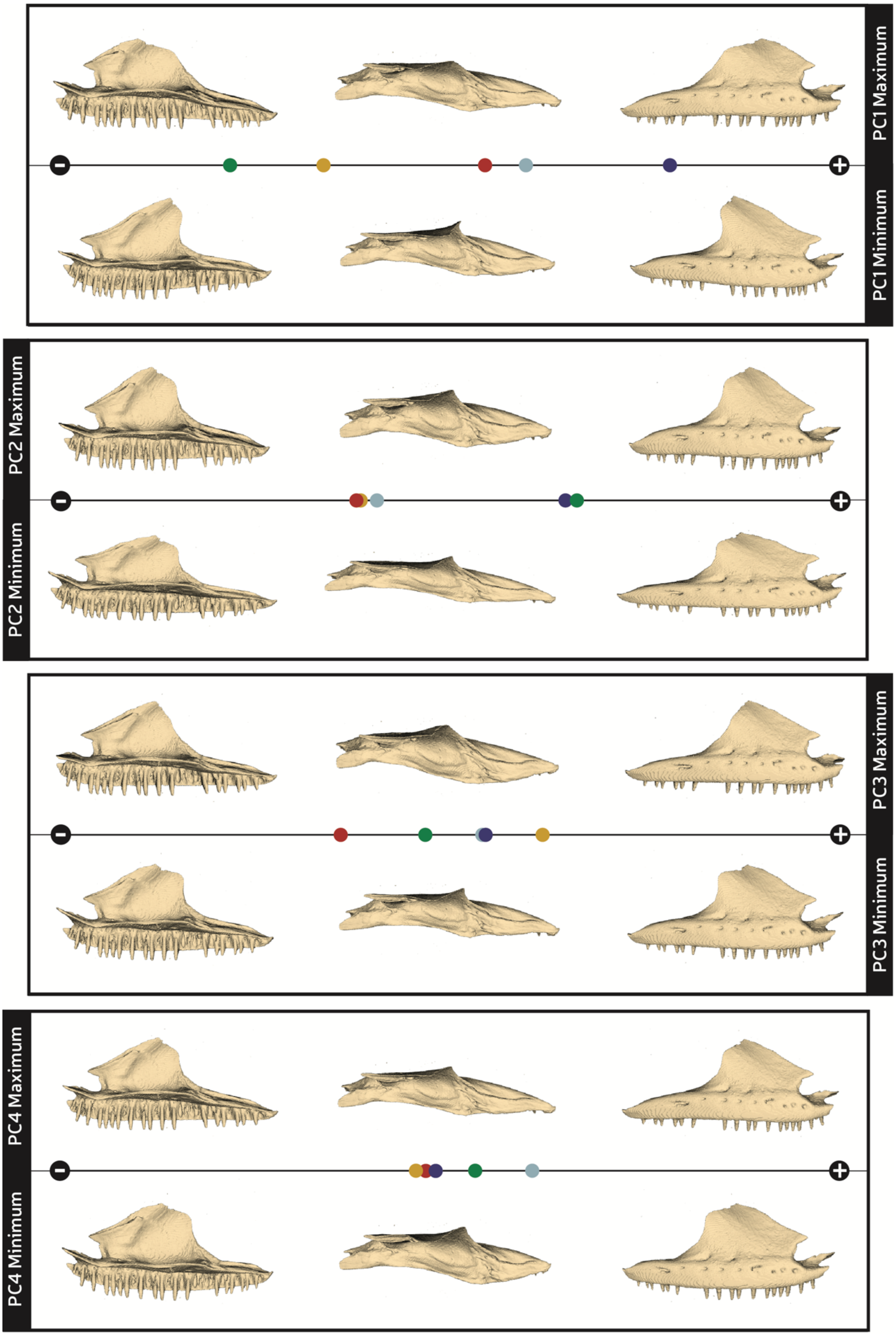

Supplementary Figure 4 Four principal axes of maxilla shape variation visualized as surface warps in medial, dorsal and lateral view (from left to right). PC axes were derived from a PCA of the symmetric component of extant diplodactylid maxilla shape. Surface warps represent the extreme maximum (+) and minimum (-) shape differences along each PC axis. Position of mean shapes for each genus are indicated (coloured circles) along each axis.

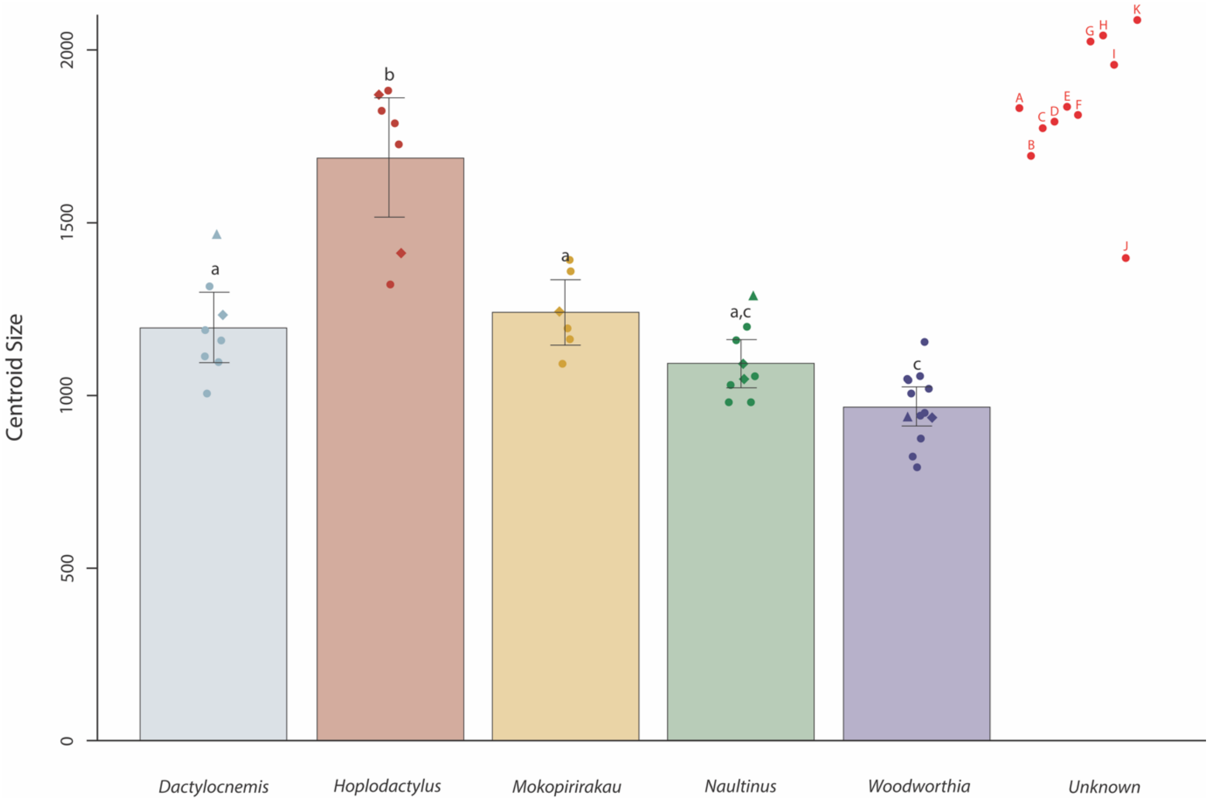

Supplementary Figure 5 Barplot of extant maxilla centroid size (coloured by genera) showing means ± SE, with points coloured by species. Genera that are significantly different are represented by a different lowercase letter (a-c). Holocene subfossil individuals are shown as red circles (A-J): Waitomo (A: AU7700, K: WO333), Wairarapa (I: S.46528.1), Tasman (G: S.38813.2; H: S.39086), North Canterbury (B: S.33703.2, C: S.33703.3, D: S.33703.4, E: S.33703.7, F: S.33703.8) and North Otago (J: VT791a).

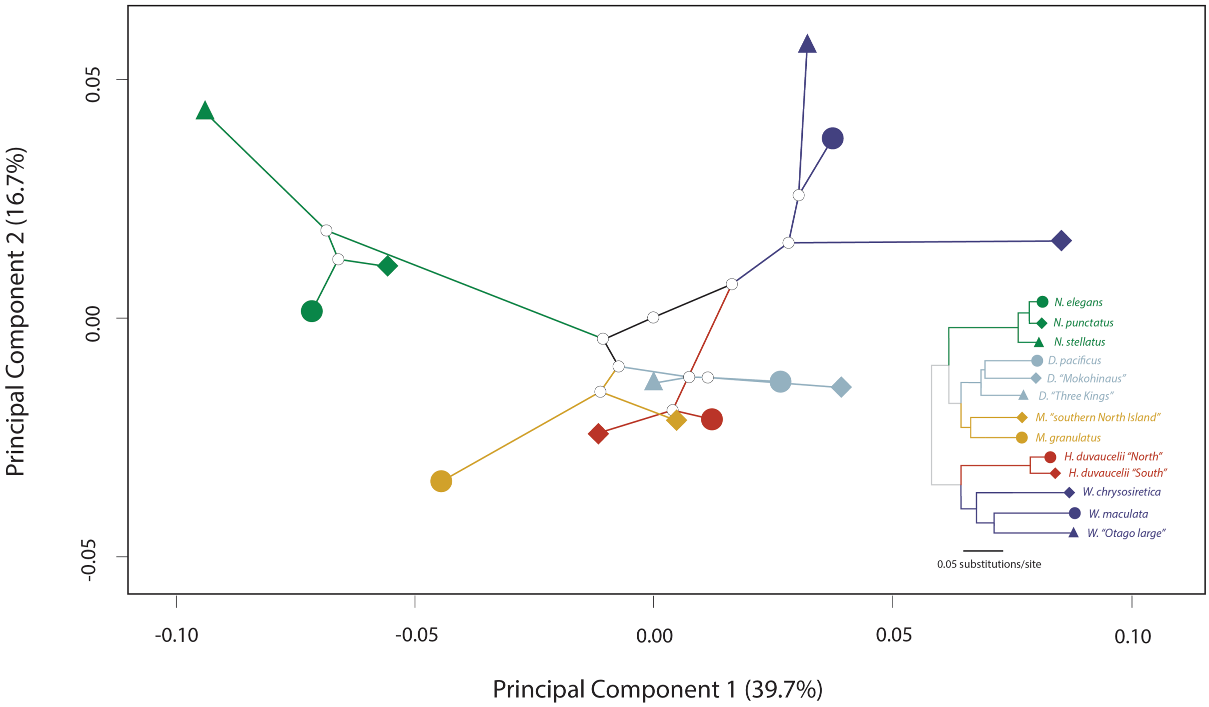

Supplementary Figure 6 Phylomorphospace representing maxilla shape variation (PC1 versus PC2) of mean symmetric shape data. Points represent described/undescribed species (mean symmetric component of left-right maxilla shape for all individuals) coloured by genus (*Dactylocnemis*: blue-grey, *Hoplodactylus*: red, *Mokopirirakau*: yellow, *Naultinus*: green, *Woodworthia*; purple). White nodes represent hypothetical maxilla shape of shared ancestors.

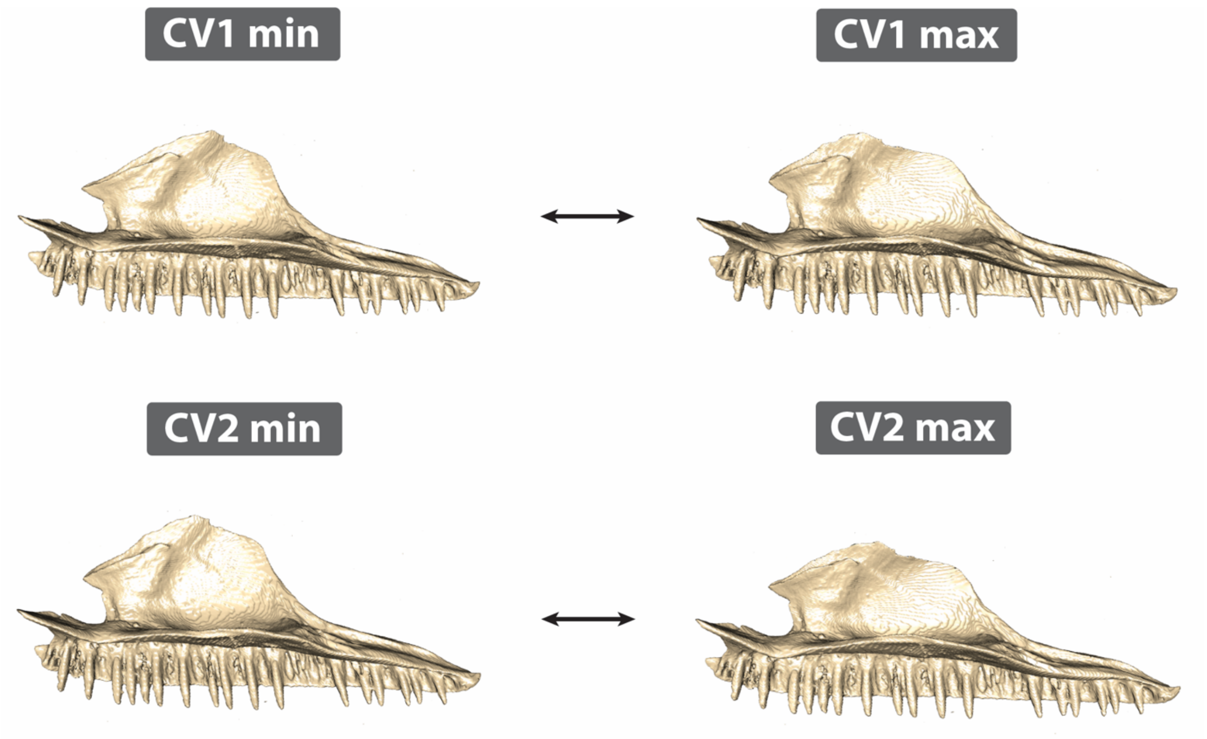

Supplementary Figure 7 Maxilla surface warps (in medial view) representing the extreme maximum and minimum shape differences along canonical variate (CV) axes 1 and 2 (see Figure 2C).

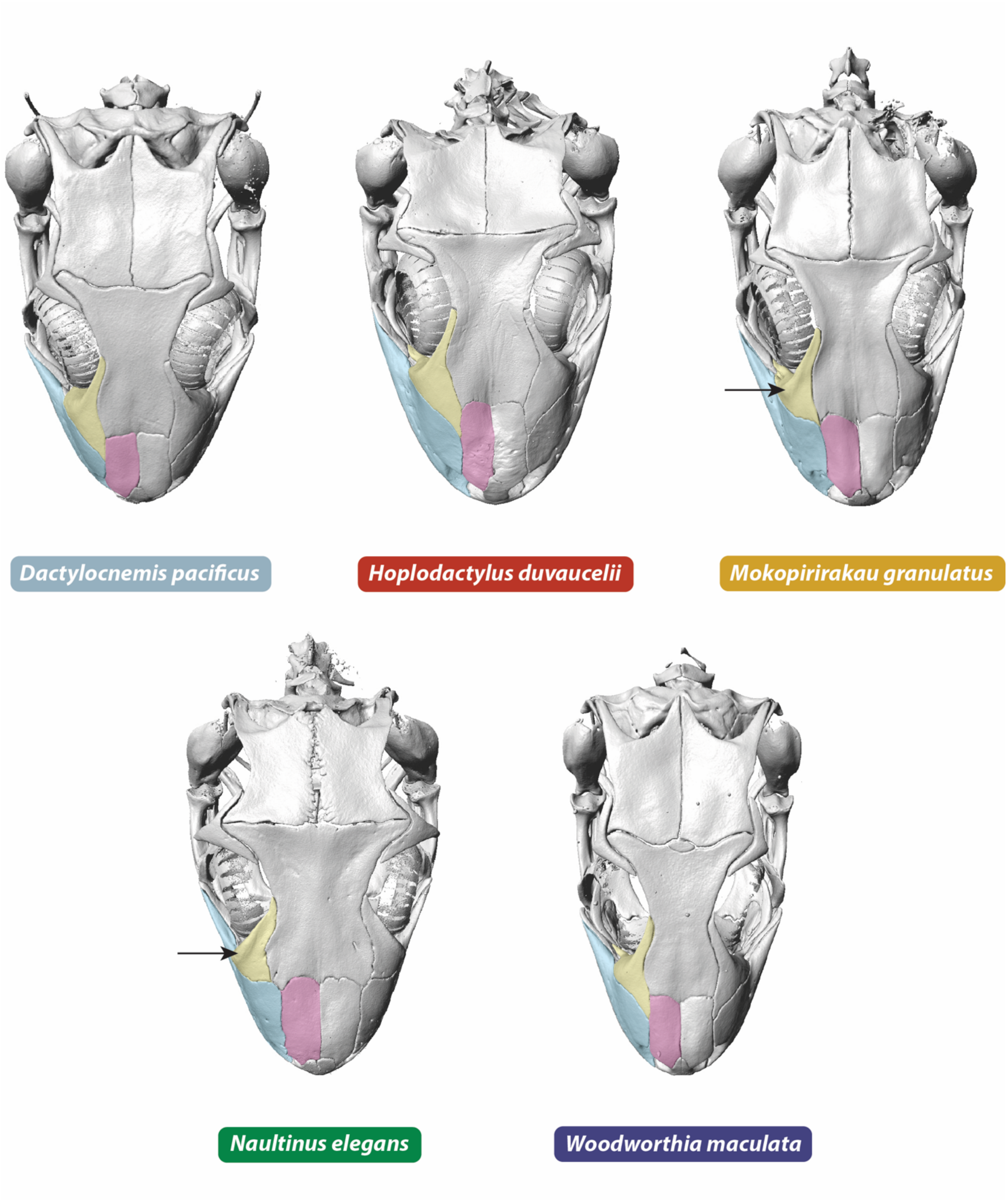

Supplementary Figure 8 Comparative surface models of diplodactylid genera skulls (in dorsal view; (42)) highlighting relative position of prefrontal (yellow), maxilla (blue-grey) and nasal (pink). Arrows indicate location of a thickened ridge along the prefrontal orbital margin (present in *Mokopirirakau* and *Naultinus*).

Supplementary Tables (S1-S8)

Supplementary Table 1 List of modern/Holocene subfossil specimens in morphometric analyses with ID (museum accession number), species-level identification, institution and collection locality. Institution abbreviations are as follows: AM: Auckland Museum; AUM: Auckland University Museum; NMNZ: Museum of New Zealand Te Papa Tongarewa; OM: Otago Museum; WCM: Waitomo Caves Museum. References associated with subfossil collection localities (of '*H.* cf. *duvaucelii*' material) are shown as superscript values: [1] (43), [2] (44), [3] (45), [4] (46), [5] (47); with estimates for deposit ages reported (where available) in thousands of years (ka).

ID - Institution	Species	Collection Locality
LH676 -AM	*Dactylocnemis pacificus*	Tarahiki I.
LH805 - AM	*Dactylocnemis pacificus*	Tarahiki I.
LH808 - AM	*Dactylocnemis pacificus*	Tarahiki I.
LH933 - AM	*Dactylocnemis* "Mokohinaus"	Mokohinau I.
LH939 - AM	*Dactylocnemis pacificus*	Tarahiki I.
LH942 - AM	*Dactylocnemis pacificus*	Tarahiki I., off Ponui I.
RE.1851 - NMNZ	*Dactylocnemis* "Three Kings"	Great Island, Three Kings Islands
RE.8596 - NMNZ	*Dactylocnemis pacificus*	Middle Island, Mercury Island
LH2942 - AM	*Hoplodactylus duvaucelii*	Whatupuke I, Hen & Chickens
LH3047 - AM	*Hoplodactylus duvaucelii*	Whatupuke I, Hen & Chickens
LH3053 - AM	*Hoplodactylus duvaucelii*	Whatupuke I, Hen & Chickens
RE.1852 - NMNZ	*Hoplodactylus duvaucelii*	The Brothers
RE.7372 - NMNZ	*Hoplodactylus duvaucelii*	Unknown Locality
RE.8590 - NMNZ	*Hoplodactylus duvaucelii*	Coppermine Island, & and Chickens
RE.8591 - AM	*Hoplodactylus duvaucelii*	South Trios Island, Cook Strait
LH3801 - AM	*Mokopirirakau granulatus*	Krippner Rd. Bush, Puhoi
LH673 - AM	*Mokopirirakau granulatus*	Titirangi
LH888 - AM	*Mokopirirakau granulatus*	Glenfield, Auckland
LH919 - AM	*Mokopirirakau granulatus*	Birkenhead, Auckland
LH920 - AM	*Mokopirirakau granulatus*	NZ
RE.8597 - NMNZ	*Mokopirirakau* "southern North Island"	Wellington
LH912 - AM	*Naultinus elegans*	Inner Gulf Islands Ecological District
LH929 - AM	*Naultinus elegans*	Inner Gulf Islands Ecological District
LH934 - AM	*Naultinus elegans*	Tamaki Ecological District
LH940 - AM	*Naultinus elegans*	Tamaki Ecological District
LH943 - AM	*Naultinus elegans*	Tamaki Ecological District
LH949 - AM	*Naultinus elegans*	North Island Ecological Region
RE.1855 - NMNZ	*Naultinus punctatus*	Whitemans Valley, Upper Hutt
RE.1856 - NMNZ	*Naultinus stellatus*	St. Arnaud, Nelson
RE.8599 - NMNZ	*Naultinus punctatus*	Wellington
LH841 - AM	*Woodworthia chrysosiretica*	Unknown, NZ
LH928 - AM	*Woodworthia maculata*	Tarahiki I.
LH935 - AM	*Woodworthia maculata*	Tarahiki I.
LH938 - AM	*Woodworthia maculata*	Tarahiki I.
LH944 - AM	*Woodworthia maculata*	Tarahiki I.
LH952 - AM	*Woodworthia maculata*	Rakitu I.
LH955 - AM	*Woodworthia maculata*	Tarahiki I.
LH990 - AM	*Woodworthia* "Otago large"	Tree I., Lake Wakatipu
RE.5286 - NMNZ	*Woodworthia maculata*	Linden, Wellington
RE.8592 - NMNZ	*Woodworthia maculata*	Cape Turakirae, Wellington
RE.8593 - NMNZ	*Woodworthia maculata*	Cape Turakirae, Wellington
RE.8594 - NMNZ	*Woodworthia maculata*	Cape Turakirae, Wellington
RE.8595 - NMNZ	*Woodworthia maculata*	Cape Turakirae, Wellington
AU7700 (A) - AUM	*Hoplodactylus cf. duvaucelii*	Little Lost World[1]
S.33703.2 (B) - NMNZ	*Hoplodactylus cf. duvaucelii*	Ardenest[2] (3 ka – Present)
S.33703.3 (C) - NMNZ	*Hoplodactylus cf. duvaucelii*	Ardenest[2] (3 ka – Present)
S.33703.4 (D) - NMNZ	*Hoplodactylus cf. duvaucelii*	Ardenest[2] (3 ka – Present)

S.33703.7 (E) - NMNZ	*Hoplodactylus cf. duvaucelii*	Ardenest[2] (3 ka – Present)
S.33703.8 (F) - NMNZ	*Hoplodactylus cf. duvaucelii*	Ardenest[2] (3 ka – Present)
S.38813.2 (G) - NMNZ	*Hoplodactylus cf. duvaucelii*	Gouland Downs[3]
S.39086 (H) - NMNZ	*Hoplodactylus cf. duvaucelii*	Takaka Hill[4] (10 ka – Present)
S.46528.1 (I) - NMNZ	*Hoplodactylus cf. duvaucelii*	Mataikona River
VT791a (J) - OM	*Hoplodactylus cf. duvaucelii*	Earthquakes[5] (1.7 ka – 1.4 ka)
WO333 (K) - WCM	*Hoplodactylus cf. duvaucelii*	Companionway Cave[1]

Supplementary Table 2 Anatomical definitions of fixed (1-15) landmarks and curves comprising semi-landmarks (C1-C4).

Landmarks	Location on Maxilla
1	Anterior-most point of the maxillary lappet
2	Anterior-most point of the anterolateral lappet
3	Most medial point of the anterolateral lappet
4	Most concave point of the embayment between 1 and 2
5	Dorsal to the anterior-most lateral foramen
6	Most concave point of the anterior palatal shelf
7	Most concave point of the nasal basin
8	Anterior-most point of the facial process
9	Apex (most dorsal point) of the facial process
10	Most dorsal point of the medial flange
11	Most medial point of the palatal shelf
12	Break in slope between the orbital margin and the posterior orbital process
13	Posterior-most point of the posterior maxillary process
14	Most ventral point of the maxillary lappet
15	Opening of the superior alveolar canal
C.1: 16-25	Along the orbital/prefrontal margin from 12 to 9
C.2: 26-35	Along the palatal shelf from 11 to 6
C.3: 36-45	Along the nasal margin from 9 to 8
C.4: 46-55	Along the palatal shelf from 11 to 13

Supplementary Table 3 Examining fluctuating asymmetry: Procrustes ANOVAs of diplodactylid maxilla centroid size (A) and shape (B) by individual, side, replicate and their interaction terms, with statistical significance assessed through 10,000 permutations. Significant results ($p < 0.05$) are indicated in bold.

(A)

	d.f.	*SS*	*MS*	R^2	*F*	*Z*	*p-value*
individual	55	0.0004923	8.9508E-06	0.76129	10.3228	9.2071	**0.0001**
side	1	0.00000039	3.931E-07	0.00061	0.4534	0.2096	0.5008
individual : side	38	0.000082	2.1579E-06	0.12681	2.4887	3.2754	**0.0006**
individual : side : replicate	83	0.00007197	8.671E-07	0.11129			
total	177	0.00064666					

(B)

	d.f.	*SS*	*MS*	R^2	*F*	*Z*	*p-value*
individual	55	1.19401	0.0217093	0.86298	6.7477	-1.011	0.8433
side	1	0.00319	0.003188	0.0023	0.9909	0.134	0.4245
individual : side	38	0.12226	0.0032173	0.08836	4.1638	38.683	**0.0001**
individual : side : replicate	83	0.06413	0.0007727	0.04635			
total	177	1.38359					

Supplementary Table 4 Procrustes distances resulting from Procrustes superimposition of the extant dataset after projection of Holocene subfossil individuals into the morphospace. Shortest Procrustes distances for each Holocene subfossil individual to extant genera (conferring shape similarity) are indicated in bold.

	Dactylocnemis	*Hoplodactylus*	*Mokopirirakau*	*Naultinus*	*Woodworthia*
Hoplodactylus	0.048				
Mokopirirakau	0.073	0.066			
Naultinus	0.103	0.091	0.067		
Woodworthia	0.064	0.081	0.11	0.12	
AU7700 (A)	0.086	**0.075**	0.116	0.112	0.077
S.33703.2 (B)	0.1	0.106	0.137	0.143	**0.088**
S.33703.3 (C)	0.085	**0.075**	0.094	0.097	0.099
S.33703.4 (D)	0.075	**0.071**	0.082	0.096	0.099
S.33703.7 (E)	**0.064**	0.065	0.081	0.098	0.092
S.33703.8 (F)	0.081	0.082	0.105	0.108	**0.075**
S.38813.2 (G)	0.074	**0.072**	0.092	0.18	0.093
S.39086 (H)	0.079	0.092	0.127	0.142	**0.065**
S.46528.1 (I)	0.077	**0.076**	0.111	0.13	0.086
VT791a (J)	0.084	**0.083**	0.111	0.107	**0.083**
WO333 (K)	**0.056**	0.061	0.099	0.112	0.063

Supplementary Table 5 Procrustes ANOVA of maxilla shape by genus, centroid size and their interaction, with statistical significance assessed through 10,000 permutations. Significant results ($p < 0.05$) are indicated in bold.

	d.f.	*SS*	*MS*	R^2	*F*	*Z*	*p-value*
genus	4	0.12723	0.03181	0.45158	9.0146	5.9143	**0.0001**
centroid size	1	0.01901	0.01901	0.06748	5.3884	2.1592	**0.0201**
genus : centroid size	4	0.01906	0.00477	0.06766	1.3507	1.9329	**0.0233**
residuals	33	0.11643	0.00353	0.41328			
total	42	0.28173					

Supplementary Table 6 Significance values for post-hoc pairwise comparison between genera maxilla shape. Significant results ($p < 0.05$) are indicated in bold.

	Dactylocnemis	*Hoplodactylus*	*Mokopirirakau*	*Naultinus*
Hoplodactylus	0.2294			
Mokopirirakau	**0.0218**	0.056		
Naultinus	**0.0001**	**0.001**	**0.0363**	
Woodworthia	**0.0147**	**0.0022**	**0.0001**	**0.0001**

Supplementary Table 7 One-way ANOVA of maxilla centroid size. Significant results (*p < 0.05*) are indicated in bold.

	d.f.	*SS*	*MS*	*F*	*p-value*
genus	4	2486579	621645	32.22	**<0.0001**
residuals	38	730774	19231		
total	42				

Supplementary Table 8 Significance values for HSD post-hoc comparison between genera maxilla size. Significant results ($p < 0.05$) are indicated in bold.

	Dactylocnemis	*Hoplodactylus*	*Mokopirirakau*	*Naultinus*
Hoplodactylus	**<0.0001**			
Mokopirirakau	0.977	**0.0001**		
Naultinus	0.536	**<0.0001**	0.2737	
Woodworthia	**0.0061**	**<0.0001**	**0.0026**	0.2526

Supplementary Table 9 Posterior probabilities of extant genera comparisons based on Mahalanobis distance using 10,000 permutations. Significant results ($p < 0.05$) are indicated in bold.

	Dactylocnemis	*Hoplodactylus*	*Mokopirirakau*	*Naultinus*
Hoplodactylus	**0.0015**			
Mokopirirakau	**0.0227**	**0.0023**		
Naultinus	**0.0001**	**0.0001**	**0.0006**	
Woodworthia	**0.0001**	**0.0001**	**0.0001**	**0.0001**

References for Supplementary Material

1. Cardini A. Lost in the other half: Improving accuracy in geometric morphometric analyses of one side of bilaterally symmetric structures. Syst Biol. 2016;65(6):1096–106. doi:10.1093/sysbio/syw043.
2. Gray JA, McDowell MC, Hutchinson MN, Jones MEH. Geometric morphometrics provides an alternative approach for interpreting the affinity of fossil lizard jaws. J Herpetol. 2017;51(3):375–82. doi:10.1670/16-145.
3. Dollion AY, Cornette R, Tolley KA, Boistel R, Euriat A, Boller E, et al. Morphometric analysis of chameleon fossil fragments from the Early Pliocene of South Africa: a new piece of the chamaeleonid history. Sci Nat. 2015;102(1–2):1–14. doi:10.1007/s00114-014-1254-3.
4. Lee MSY, Hutchinson MN, Worthy TH, Archer M, Tennyson AJD, Worthy JP, et al. Miocene skinks and geckos reveal long-term conservatism of New Zealand's lizard fauna. Biol Lett. 2009;5(6):833–7. doi:10.1098/rsbl.2009.0440.
5. Worthy TH. A review of the fossil record of New Zealand lizards. In: New Zealand Lizards. Springer International Publishing; 2016. p. 65–86. doi:10.1007/978-3-319-41674-8_3.
6. Nielsen S V, Bauer AM, Jackman TR, Hitchmough RA, Daugherty CH. New Zealand geckos (Diplodactylidae): Cryptic diversity in a post-Gondwanan lineage with trans-Tasman affinities. Mol Phylogenet Evol. 2011;59(1):1–22.
7. Morgan-Richards M, Hinlo AR, Smuts-Kennedy C, Innes J, Ji W, Barry M, et al. Identification of a rare gecko from North Island New Zealand, and genetic assessment of its probable origin: A novel mainland conservation priority? J Herpetol. 2016;50(1):77–86. doi:10.1670/13-128.
8. Ljubisavljević K, Urošević A, Aleksić I, Ivanović A. Sexual dimorphism of skull shape in a lacertid lizard species (*Podarcis* spp., *Dalmatolacerta* sp., *Dinarolacerta* sp.) revealed by geometric morphometrics. Zoology. 2010;113(3):168–74.
9. Sanger TJ, Sherratt E, McGlothlin JW, Brodie ED, Losos JB, Abzhanov A. Convergent evolution of sexual dimorphism in skull shape using distinct developmental strategies. Evolution. 2013;67(8):2180–93. doi:10.1111/evo.12100.
10. Kaliontzopoulou A, Carretero MA, Adams DC. Ecomorphological variation in male and female wall lizards and the macroevolution of sexual dimorphism in relation to habitat use. J Evol Biol. 2015;28(1):80–94. doi:10.1111/jeb.12540.
11. Evans S. The skull of lizards and Tuatara. In: Gans C, Gaunt A, editors. Biology of the Reptilia. New York: Ithaca SSAR; 2008. p. 1–347.
12. Ledesma DT, Scarpetta SG. The skull of the gerrhonotine lizard *Elgaria panamintina* (Squamata: Anguidae). PLoS One. 2018;13(6):e0199584. doi:10.1371/journal.pone.0199584.
13. Schindelin J, Arganda-Carreras I, Frise E, Kaynig V, Longair M, Pietzsch T, et al. Fiji: An open-source platform for biological-image analysis. Nat Methods. 2012;9(7):676–82. doi:10.1038/nmeth.2019.
14. Arnqvist G, Mårtensson T. Measurement error in geometric morphometrics: Empirical strategies to assess and reduce its impact on measures of shape. Acta Zool Acad Sci Hungaricae. 1998;44(2):73–96.
15. R Development Core Team. R: a language and environment for statistical computing. Vienna; 2019.
16. Adams DC, Collyer M, Kaliontzopoulou A. geomorph: Software for geometric morphometric analyses. https://cran.r-project.org/web/packages/geomorph/index.html. 2020;

17. Schlager S. Morpho: calculations and visualizations related to geometric morphometrics. 2016;
18. Rohlf FJ, Slice D. Extensions of the procrustes method for the optimal superimposition of landmarks. Syst Zool. 1990;39(1):40. doi:10.2307/2992207.
19. Klingenberg CP, Barluenga M, Meyer A. Shape analysis of symmetric structures: Quantifying variation among individuals and asymmetry. Evolution. 2002;56(10):1909–20. doi:10.1111/j.0014-3820.2002.tb00117.x.
20. Bookstein FL. Landmark methods for forms without landmarks: Morphometrics of group differences in outline shape. Med Image Anal. 1997;1(3):225–43.
21. Dryden I, Mardia K. Statistical analysis of shape. Chichester, UK: Wiley; 1998.
22. Dickson B V, Sherratt E, Losos JB, Pierce SE. Semicircular canals in *Anolis* lizards: ecomorphological convergence and ecomorph affinities of fossil species. R Soc Open Sci. 2017;4(10):170058. doi:10.1098/rsos.170058.
23. Drake AG, Klingenberg CP. Large-scale diversification of skull shape in domestic dogs: Disparity and modularity. Am Nat. 2010;175(3):289–301.
24. Bookstein FL. Principal warps: Thin-plate splines and the decomposition of deformations. IEEE Trans Pattern Anal Mach Intell. 1989;11(6):567–85.
25. Kraatz B, Sherratt E. Evolutionary morphology of the rabbit skull. PeerJ. 2016;2016(9):e2453. doi:10.7717/peerj.2453.
26. Adams DC. A method for assessing phylogenetic least squares models for shape and other high-dimensional multivariate data. Evolution. 2014;68(9):2675–88. doi:10.1111/evo.12463.
27. Goodall C. Procrustes methods in the statistical analysis of shape. J R Stat Soc Ser B. 1991;53(2):285–321. doi:10.1111/j.2517-6161.1991.tb01825.x.
28. Collyer ML, Sekora DJ, Adams DC. A method for analysis of phenotypic change for phenotypes described by high-dimensional data. Heredity (Edinb). 2015;115(4):357–65. doi:10.1038/hdy.2014.75.
29. Adams DC, Collyer ML. Phylogenetic ANOVA: Group-clade aggregation, biological challenges, and a refined permutation procedure. Evolution. 2018;72(6):1204–15. doi:10.1111/evo.13492.
30. Copenhaver M, Holland B. Computation of the distribution of the maximum studentized range statistic with application to multiple significance testing of simple effects. J Stat Comput Simul. 1988;30(1):1–15. doi:10.1080/00949658808811082.
31. Adams D. A generalized K statistic for estimating phylogenetic signal from shape and other high-dimensional multivariate data. Syst Biol. 2014;63(5):685–97. doi:10.1093/sysbio/syu030.
32. Blomberg SP, Garland T, Ives AR. Testing for phylogenetic signal in comparative data: Behavioral traits are more labile. Evolution. 2003;57(4):717–45. doi:10.1111/j.0014-3820.2003.tb00285.x.
33. Chong N. Phylogenetic analysis of the endemic New Zealand gecko species complex *Hoplodactylus pacificus* using DNA sequences of the 16S rRNA gene. Victoria University, Wellington, New Zealand.; 1999.
34. Albrecht GH. Multivariate analysis and the study of form, with special reference to canonical variate analysis. Am Zool. 1980;20:679–93. doi:10.1093/icb/20.4.679.
35. Strauss R. Discriminating groups of organisms. In: Elewa A, editor. Morphometrics for non morphometricians. Berlin, Germany: Springer; 2010. p. 73–91.
36. Mitteroecker P, Bookstein F. Linear discrimination, ordination, and the visualization of selection gradients in modern morphometrics. Evol Biol. 2011;38(1):100–14. doi:10.1007/s11692-011-9109-8.
37. Wilson SR. On comparing fossil specimens with population samples. J Hum Evol.

1981;10(3):207–14.
38. Albrecht GH. Assessing the affinities of fossils using canonical variates and generalized distances. Hum Evol. 1992;7(4):49–69. doi:10.1007/BF02436412.
39. Huberty C, Olejnik S. Applied MANOVA and discriminant analysis. New Jersey, USA: Wiley; 2006.
40. Klecka W. Discriminant Analysis. Newbury Park: Sage Publications; 1980.
41. Campbell NA. Some aspects of allocation and discrimination. In: Multivariate Statistical Methods in Physical Anthropology. Springer Netherlands; 1984. p. 177–92. doi:10.1007/978-94-009-6357-3_12.
42. Paluh DJ, Bauer AM. Phylogenetic history, allometry and disparate functional pressures influence the morphological diversification of the gekkotan quadrate, a keystone cranial element. Biol J Linn Soc. 2018;125(4):693–708. doi:10.1093/BIOLINNEAN/BLY147.
43. Worthy TH. Osteological observations on the larger species of the skink *Cyclodina* and the subfossil occurrence of these and the gecko *Hoplodactylus duvaucelii* in the North Island, New Zealand. New Zeal J Zool. 1987;14(2):219–29. doi:10.1080/03014223.1987.10422992.
44. Worthy TH, Holdaway RN. Quaternary fossil faunas, overlapping taphonomies, and palaeofaunal reconstruction in North Canterbury, South Island, New Zealand. J R Soc New Zeal. 1996;26(3):275–361. doi:10.1080/03014223.1996.9517514.
45. Worthy TH. A fossil vertebrate fauna accumulated by laughing owls (Sceloglaux albifacies) on the Gouland Downs, northwest Nelson, New Zealand. Notornis. 2001;48:225–33.
46. Worthy TH, Holdaway RN. Quaternary fossil faunas from caves in Takaka Valley and on Takaka Hill, northwest Nelson, South Island, New Zealand. J R Soc New Zeal. 1994;24(3):297–391. doi:10.1080/03014223.1994.9517474.
47. Worthy TH. Quaternary fossil faunas of Otago, South Island, New Zealand. J R Soc New Zeal. 1998;28(3):421–521. doi:10.1080/03014223.1998.9517573.
